# The long-term effects of a prevention program on the number of critical incidents and sick leave days

**DOI:** 10.1186/s13033-018-0250-y

**Published:** 2018-11-21

**Authors:** V. Isaak, D. Vashdi, O. Steiner-Lavi

**Affiliations:** 1 The Division of Public Administration and Policy, School of Political Sciences, University of Haifa, Mount Carmel, 31905 Haifa, Israel; 2 Management Faculty, MLA The College for Academic Studies, Or Yehuda, Israel

**Keywords:** Wellbeing, Violence at work, Intervention program refreshers, Public sector mental healthcare

## Abstract

**Background:**

This study explores the effectiveness of refresher training sessions of an intervention program at reducing the employees’ risk of injury due to patient violence in forensic psychiatric hospital.

**Methods:**

The original safety intervention program that consisted of a 3 days’ workshop was conducted in the maximum—security ward of a psychiatric hospital in Israel. Ever since the original intervention, annual refreshers were conducted highlighting one of the safety elements covered in the original intervention. The study examines the effect of the intervention program along with the refreshers over a period of 10 years in four wards.

**Results:**

Analysis of the data demonstrates that beyond the initial reduction following the original intervention, refreshers seem to have an additional positive long-term effect, reducing both the number of violent incidents and the number of actual employee injuries in forensic psychiatric hospital.

**Conclusions:**

We conclude that such an intervention program followed by refresher training would promote employees’ wellbeing. A healthy work environment is part of management’s commitment to improve employee wellbeing at the workplace.

## Introduction

### Wellbeing at work

Many studies have shown the correlation between positive organizational processes, job satisfaction and wellbeing [1–4]. Similarly, research has demonstrated that wellbeing at work is an important factor determining organizational success (e.g. [5–7]). On the flipside, research has also shown the detrimental effects of lack of wellbeing on organizational productivity and performance [8]. When employees feel unsafe or mistreated they are likely to be tardy, absent or even to resign [9], influencing organizational outcomes. One factor influencing employee wellbeing is the extent to which the work environment is aggressive or violent [10]. Thus, many policies and interventions are being put in place in organizations to try to minimize such aggression or violence [11, 12]. Yet little is known regarding the long-term effects of these interventions and whether their affect is sustainable. In accordance, the aim of the current study is to examine the long-term effect of refreshers of a safety intervention program on reducing violence at work and creating a healthy work environment.

### Violence in the mental health system

Mental patient violence has been described as “The dark side of mental disorders” [13, 14] with high rates of violent incidents in psychiatric institutions. Studies in the United States, Canada, Belgium, and Australia show that 26–56% of hospital and community agency staff who treat psychiatric patients have been victims of assault [15, 16]. The concomitant implications of these incidents are physical pain, emotional suffering and impaired therapeutic relations as well as high financial costs for the organization [17, 18]. Hillbrand et al. [19] reported that over 2% of maximum-security forensic hospital budgets in the United States were directed to salaries for employees hired to replace personnel absent due to patient-inflicted injuries and there are indications that the magnitude of workplace violence is steadily increasing [20].

In most cases, nurses are the victims of such violence [21–24]. A recent study in Poland found higher rates of violent incidents against psychiatric nurses compared to nurses in other medical disciplines [20]. In the United States, reports suggest that one in four psychiatric nurses are assaulted by patients and require work absence each year [25]. An Australian study, conducted on closed psychiatric wards, found that 78% of the victims of patient assaults were nurses, 4% were physicians, 2% were psychologists, and 2% were social workers [16]. The difference was explained by the nurses’ constant attendance to the patients. In addition, nurses are those who provide primary treatment for violent patients, especially during outbursts [22, 24, 26]. As a result, nurses in forensic units describe their work as an ongoing conflict between their desire to assist patients, as expected of nurses, and their need to avoid harm by patients [27].

As a result of this phenomena, for the last two decades, the United States Occupational Safety and Health Administration (OSHA) has stressed psychiatric institution are responsible for maintaining safety standards toward minimizing occupational injuries [17, 18]. Alongside these policies various interventions aimed at reducing violence toward employees, either specifically in psychiatric facilities or generally in workplaces have been proposed [27, 28].

### Intervention programs to reduce violence against employees

Several different interventions have been proposed and studied with the aim of minimizing employee injuries. These interventions include structured feedback after violent events and violence management teams [28, 29]. Studies have assessed these interventions in hospitals reporting their success [29–33]. Other interventions have focused on staff training toward managing patient aggression [34, 35]. Finally, designing environments with fewer risk factors (e.g. ensuring patients and staff can be seen at all times) is another intervention that has been implemented and studied [11, 12]. Recently, Isaak et al. [36] proposed a new intervention combining many of the aspects learnt from previous interventions. They found that their specific intervention enhanced a safety climate in the relevant hospital wards and showed a reduction in both the number of aggressive incidents towards employees and in the number of employee injuries.

Thus, most of these studies focused on how these interventions reduce violent incidents or employee injuries. Yet these studies did not examine the long-term effects of these interventions, mostly conducting a “before and after” research program. In the current study, we examine if annual refresher sessions conducted based on the intervention proposed in Isaak et al. [36] may have a prolonged impact continuing to reduce injuries and violent incidents.

### The impact of refresher training sessions

Based on learning theories [37–39] skills tend to decay with time leading to the need for appropriate retraining methods. Such relearning is usually shorter in duration than the original learning period with procedural skills demanding longer practice time than psychomotor skills. Such theories have been proven in the healthcare sector in studies showing (for example) the importance of CPR refresher training [40]. Yet, there seems to be much less research regarding the importance of refreshing learning that has to do with enhancing a safety climate. Research is needed to examine whether refreshing skills associated with more soft skills such as better communication or after event debriefings will also serve to further minimize violence towards employees.

## Methods

We returned to the same hospital where the study conducted by Isaak et al. [36] took place. In their study, they examined the effect of a 3 days intervention called the “Getting Home Safely” in a Mental Health Clinic’s (MHC) maximum-security unit in Israel. The intervention program was delivered to the entire staff of the forensic psychiatry departments [36].

The intervention program is a 3 days program including the following:

- Day 1—Personal safety: Participants learn how to avoid dangerous situations, self-defense skills, and methods for safely restraining patients.
- Day 2—Participants learn how to use tools for better inter-staff communication.
- Day 3—Organizational learning. Participants learn how to conduct incident investigations after adverse events based on the model used by the IDF (Israeli Defense Force).

In order to maintain the outcomes of the intervention program, since 2009 refresher sessions have been conducted on a regular basis each year. The staff decides which module day (i.e., personal safety, communication, or organizational learning) to emphasize each year. Each department manager receives notification of the need for a refresher in his department from his supervisor. The department manager conducts a survey among all the employees, and based on their choice, the selected topic is refreshed.

The refresher sessions are continuing and the most recent one was conducted in 2017 in all the four units of the forensic psychiatry departments.

## Sample and procedure

Based on organizational reports, we received additional data presented in Isaak et al. [36] regarding violent incidents in the MHC’s four maximum-security units in the 10 years following the initial intervention. In addition, we received absenteeism days regarding all staff for the whole period from 2007, when the intervention was initially conducted, to 2017. In the four maximum-security units there are 112 employees (according to the HR division reports, there is a fixed number of employees in this four units with no changes over the period between 2007 and 2017) (Table 1).

**Table 1:** Demographic characteristics of hospital staff

Gender		Sector		Education		Seniority in unit (years)		Seniority in organization (years)	
Male	47.4%	Doctor	7.14%	High school	43.1%	Up 1	18.6%	Up 1	10.6%
Female	52.6%	Nurse	71.43%	Bachelor degree	28.1%	1–10	42.3%	1–10	29.6%
		Other^a^	21.43%	Master degree	15.6%	11–20	24.4%	11–20	37.3%
				Other^b^	13.1%	21–30	7.7%	21–30	16.9%
						31–40	7.1%	31–40	5.6%

^a^ Other = psychologists, social workers, occupational therapists, maintenance workers, and secretaries

^b^ Other = psychiatric graduate nursing course

## Research variables

### Number of incidents

Aggressive or violent incidents were defined as cases in which a patient physically attacked a staff member. The data was drawn from internal reports submitted to the Risk Management Director.

### Number of absenteeism days

The number of days in which employees took a day of absence in each ward was examined using internal reports to the Risk Management Director.

## Results

As can be seen in Table 2, beyond the decrease already shown in Isaak et al. [36], there is a rather steady decrease in the number of violent incidents throughout the maximum security unit from 2013 to 2017. As the refresher sessions started in 2009 and have been conducted annually, over the 8 years it seems that these refresher sessions are important as the rate of incidents is kept low in comparison to the pre-intervention years.

**Table 2:** The number of aggressive incidents towards staff before and after the intervention in all four forensic units

Before the intervention program					After the intervention program									
Year	2004	2005	2006	2007	2008	2009	2010	2011	2012	2013^a^	2014	2015	2016	2017
Number of incidents^b^	13	19	55	36	26	18	8	14	12	13	10	6	16	13

^a^ All data up until this point was presented in Isaak et al. [36]

^b^ Incidents include accident with and without physical injury

Table 3 presents decreased absenteeism days. In 2006, prior to the intervention program, the injured led to 797 absence days in total at the four units. After the intervention program had been conducted, following refresher training every year, we can see a reduction of absence days that represents a long-term effect (leading to 176 absence days in 2017).

**Table 3:** Number of absenteeism days

Before the intervention program					After the intervention program									
Year	2004	2005	2006	2007	2008	2009	2010	2011	2012	2013	2014	2015	2016	2017
Number of absence days	104	410	797	519	381	317	301	214	247	245	213	198	189	176
Loss of personel^a^	0.47	1.86	3.6	2.3	1.73	1.44	1.36	0.97	1.12	1.11	0.96	0.9	0.85	0.8

The standards of yearly working days for employee in the maximum security unit is 220 days per person

^a^ Loss of personnel = absent days per year: 220

## Discussion

While previous research has shown that the “Getting Home Safely” intervention succeeded in restoring the sense of a healthy work environment for employees at the Mental Health Center, and significantly reduced the scope of violence in the wards, the current research makes an additional contribution by showing the positive long term effects of refresher sessions. It seems that while interventions may be effective as a single training, people are likely to need refreshers to mitigate skill decay. Thus, such interventions should be followed up as they not only refresh the employee’s memory but they demonstrate that a healthy work environment is part of the management’s commitment to maintain employees’ wellbeing at work. These results are consistent with previous findings that refresher training can improve the employees’ skills [37–39]. Such interventions may allow management to create and maintain a healthy work environment [4] that enhances the emotional wellbeing [41], as well of all employees [42].

“Getting Home Safely” succeeded in restoring the sense of a healthy work environment for employees at the Sha’ar Menashe Health Center and significantly reduced the incidence of violence in the wards. Reduced patient violence provides a healthy work environment that leads to a high level of wellbeing [4].

This study has several limitations. First, only four units participated in the study and all underwent the intervention. Given the small sample and absence of a control group, our findings should not be considered in isolation, but as a part of a study supporting the concept that intervention programs can reduce violence. Second, we cannot prove that the specific content of the intervention program was the direct reason for the reported outcome. Another program may have been similarly effective. What we can see from our results is that the violence was reduced and remained at a consistent level over the past decade. Furthermore, this study focused on violence within the workplace of a forensic hospital. It may be difficult to extrapolate these findings to other workplace settings, including other areas within the health sector.

Future research should be conducted to study the effects of intervention programs such as ours in a variety of psychiatric and other healthcare organizations. We recommend a future study to compare our intervention program to others that focus on violence and their impact on wellbeing at work.
